# Evaluation of the auditory brainstem response test in patients with type 2 diabetes mellitus

**DOI:** 10.22088/cjim.15.3.527

**Published:** 2024-08-01

**Authors:** Mahbubeh Sheikhzadeh, Fereshteh Bagheri, Mohammad Ali Bayani, Milad Kami, Mohsen Monadi

**Affiliations:** 1 Department of Audiology, School of Rehabilitation Sciences, Babol University of Medical Sciences, Iran; 2 Clinical Research Development Unit of Rouhani Hospital, Department of Internal Medicine, Babol University of Medical Sciences, Babol, Iran; 3 Department of Medicine, Babol University of Medical Sciences, Iran

**Keywords:** Hearing loss, Diabetes mellitus, Auditory brainstem response, Interpeak latencies, Pure-tone audiometry

## Abstract

**Background:**

Hearing loss is an unknown complication of diabetes mellitus (DM). The aim of this study was to evaluate hearing function using auditory brainstem response (ABR) in diabetic patients.

**Methods:**

The present case-control study was performed on thirty diabetic patients as a case group and thirty healthy individuals as a control group. Baseline demographic information, HbA1c level, and duration of diabetes were obtained from all diabetic patients. In all subjects, the ABR and pure-tone audiometry (PTA) tests were performed and the results were analyzed using the t-test and logistic regression.

**Results:**

The absolute latency of I was significantly lower in diabetes patients. The absolute latency of III and the interpeak latencies (IPL) I-III were significantly higher in diabetic patients. No significant relationship was noticed in the absolute latency of V and the IPL I-V among diabetic patients in the right and left ears (P>0.05).

**Conclusion:**

The results of this study suggested that diabetes may cause central auditory dysfunction manifested on the absolute latency of III, the IPL I-III and III-V.

Diabetes mellitus (DM) with hyperglycemia is the consequence of impaired insulin secretion and insulin function or both of them (1). Type 2 diabetes also called non-insulin-dependent diabetes or adult-onset diabetes includes people who are insulin resistant or insulin-deficient (2). The global prevalence of DM is extremely high and is projected to increase from 382 million in 2013 to 592 million in 2035 (3). The studies have shown that hearing problems (average hearing threshold greater than 25 dB in the worse ear) are an unknown complication of DM. The prevalence varies from 34. 4 to 60.2%in different studies (4, 5).

 Clinical features include sensory-neural hearing loss similar to the age-related hearing loss (6). The pathogenesis of morphologic disorders in type 2 diabetes is directly related to hyperglycemia, but there are increases in diffuse thickness in the cochlear bacillary membrane, although has not yet been confirmed (7). Changes in the vascular endothelium and contraction of the smaller vessels of the inner ear lead to hypoxia and hearing loss (8, 9). 

The most common complication of diabetes is neuropathy, which can lead to a delay in the excitatory potential in the central pathways (10). Until diabetic neuropathy develops, diabetics have normal hearing function. However, once nerve damage sets in, this leads to progressive hearing loss with subsequent hearing loss (11). The auditory brainstem response (ABR) is a simple and non-invasive method for detecting early disorders of the auditory nerve and central nervous system pathways. 

The ABR test is the recording of the simultaneous response of a large number of neurons in the lower part of the auditory pathway and can electrophysiologically reveal any lesion from the auditory nerve to the brainstem to detect subclinical types and central neuropathy in diabetic patients (12).

Involvement of the cochlea and eighth nerve has been observed in the progressive sensory-neural hearing loss in diabetic patients (13). Previous studies have shown that the sensory-neural hearing loss occurs at higher frequencies in diabetes patients (14-16). However, the relationship between DM and hearing loss remains controversial (17). This study was performed to find possible involvement of the brain stem among the diabetic patients. Therefore, the aim of the present study was to evaluate the ABR test in the diabetic patients.

## Methods

This study was performed on thirty diabetic patients (30-55 years old) that referred to the Endocrinology and Audiology Clinics of Ayatollah Rouhani Hospital. Thirty healthy individuals were also selected as a control group. Subjects with the history of ear problems, long-term noise exposure, ototoxic medications, history of stroke, head injury, surgery on the ear, family history of deafness, and patients treated with medications that may affect central nervous system function (e.g., methyldopa, reserpine, phenytoin, antipsychotics, antidepressants) were excluded from the study. 

Information on age, gender, last HbA1c level, and duration of diabetes was collected from all diabetic patients. Otoscopy (Richter, German) and low-frequency tympanometry (MADSEN zodiac 901, 226Hz) were performed to rule out any perforation of the tympanic membrane, infection, and other middle ear diseases. Then, the pure-tone audiometry (PTA) (MADSEN Astera) was performed at frequencies of 250-8000 Hz. All subjects with the hearing thresholds more than 25 DbHL excluded from the study.

The ABR (Integrity,Vivosonic Inc.) responses were recorded using the click stimulus and the intensity levels of click were 20-70 dBnHL. Then the absolute latencies of I, III and V, the amplitude ratio of V/I, the interpeak latency (IPL) I-V, I-III, III-V were collected. 

**Statistical analysis: **Data were collected separately for each ear. Comparison between groups was performed using the t-test and linear regression with a confidence level of 95%. All data were analyzed using Statistical Package for the Social Sciences 24 (SPSS, Inc., Chicago, IL, USA; IBM Corp., Armonk, NY, USA).

## Results

32 (53.3%) of the sixty participants in this study were women, including 17 (56.6%) with diabetes and 15 (50%) without diabetes. Twenty-eight (46.7%) of the sixty subjects were men, including 13 (43.3%) with diabetes and 15 (50%) without diabetes. The gender distribution was similar between the two groups.

 21 (70%) of diabetic patients were treated with tablets and 9 (30%) with insulin. 

Sixteen (53.3%) of diabetes patients had duration of disease less than 5 years and 14 (46.7%) of diabetic patients had duration of disease more than 5 years. The mean total duration of diabetes in all diabetic patients was 7.03 ± 6.41 years. The mean HbA1c of the diabetic patients was 7.07± 0.80. The parameters of the ABR test were evaluated using the t-test between two groups to investigate the relationship between diabetes and the ABR parameters. The results are shown in table 1.

The results of the above table indicated that the parameters related to the absolute latency of I, III, and IPL I-III showed a significant difference between the two groups. The absolute latency of I was lower in the diabetic patients’ group than in the control group (p<0.05) and the absolute latency of III in the control group was lower than in the diabetic patients’ group (p<0.05). The IPL I-III in the diabetic patients’ group was greater than in the control group (p<0.05). 

There was no significant difference in the absolute latency of V and the IPL I-V between the two groups in both ears, but there was a significant difference in the V/I ratio between the two groups in both ears (p<0.05). A significant difference in the IPL III-V was observed between the two groups in the right ear (p<0.05), while there was no significant difference in the left ear. A significant difference was observed for the PTA between the two groups in the left ear (p<0.05), while no significant difference was observed for the PTA between the two groups in the right ear. Linear regression test was performed for each parameter to evaluate the variables of diabetes, gender, and age. The results are illustrated in table 2. 

**Table 1:** Comparison of the ABR parameters between the diabetes patients and control groups

**Parameter**		**Diabetes patients ** **group (mean±SD)**	**Control group** **(mean±SD)**	***P-value***
**Wave I**	**right**	1.39±0.15	1.51±0.04	<0.001
	**left**	1.40±0.19	1.52±0.05	0.002
**Wave III**	**right**	3.64±0.22	3.46±0.05	<0.001
	**left**	3.60±0.22	3.49±0.04	0.007
**Wave V**	**right**	5.49±0.36	5.60±0.11	0.14
	**left**	5.53±0.34	5.61±0.12	0.19
**IPL I-III**	**right**	2.25±0.21	1.95±0.07	<0.001
	**left**	2.19±0.16	1.96±0.06	<0.001
**V/I ratio**	**right**	4.43±5.92	1.93±0.27	0.024
	**left**	6.05±9.24	1.98±0.32	0.019
**IPL I-V**	**right**	4.01±0.52	4.08±0.11	0.46
	**left**	4±0.54	4.10±0.13	0.31
**IPL III-V**	**right**	1.93±0.45	2.14±0.11	0.019
	**left**	1.96±0.45	2.13±0.12	0.061
**PTA**	**right**	12.53±6.46	10.16±2.06	0.061
	**left**	12.87±6.38	9±2.03	0.002

IPL: interpeak latency, PTA: pure tone average, SD: Standard Deviation.

**Table 2:** Linear regression test for the absolute latency of I, III, and V

		**Non-standard coefficient**	**Standard coefficient**	***P-value***	**95% confidence interval**	
					**Lower limit**	**Upper limit**
**Latency wave I right ear**	**Diabetic**	-0.116	-0.457	<0.001	-0.178	-0.054
	**Sex**	0.027	0.107	0.37	-0.033	0.088
	**age**	-0.017	-0.063	0.60	-0.082	0.048
**Latency wave I left ear**	**Diabetic**	-0.106	-0.34	0.010	-0.184	-0.027
	**Sex**	0.046	0.147	0.23	-0.031	0.123
	**age**	-0.045	-0.141	0.27	-0.128	0.037
**Latency wave III right ear**	**Diabetic**	0.175	0.477	<0.001	0.086	0.263
	**Sex**	0.008	0.021	0.85	-0.079	0.094
	**age**	0.036	0.095	0.43	-0.056	0.129
**Latency wave III left**	**Diabetic**	0.123	0.357	0.008	0.034	0.212
	**Sex**	0.062	0.178	0.161	-0.025	0.149
	**age**	0.001	0.004	0.97	-0.092	0.095
**Latency wave V right ear**	**Diabetic**	-0.104	-0.190	0.16	-0.252	0.045
	**Sex**	0.073	0.134	0.31	-0.072	0.218
	**age**	0.016	0.028	0.84	-0.140	0.171
**Latency wave V left ear**	**Diabetic**	-0.077	-0.150	0.281	-0.220	0.065
	**Sex**	0.034	0.065	0.627	-0.105	0.173
	**age**	-0.030	-0.056	0.691	-0.179	0.120

A significant relationship was found between the absolute latency of I and III among diabetes patients in both ears. The absolute latency of I and III in the diabetic patients’ group was lower than the control group, but no significant relationship was found between the absolute latency of V in both ears based on the linearity regression. There was no significant relationship between the absolute latencies of I, III, and V with gender and age.

Linear regression test indicated a significant relationship between the IPL I-III and diabetes in both ears (p<0.05). There was a significant relationship between the I/V ratio and diabetes only in the right ear (p<0.05). No significant relationship was seen between IPL I-V and diabetes in both ears. There was no significant relationship between IPL I-III, I-V and I/V ratio with gender and age. The results are represented in table 3.

The linear regression test demonstrated a significant relationship between IPL III-V and diabetes only in the right ear (p<0.05). In addition, there was a significant relationship between the PTA and diabetes only in the left ear (p<0.05). There was no significant relationship between IPL III-V and the PTA with age and gender. The results are displayed in table 4.

The T-test was performed to investigate the relationship between the duration of diabetes and the parameters of the ABR test. The results are presented in Table 5. The results of the above table revealed that there is no significant relationship between the duration of diabetes more than 5 years and the parameters of the ABR test and the PTA (p>0.05). There was also no significant relationship between the duration of diabetes less than 5 years and the parameters of the ABR and the PTA (p>0.05). 

**Table 3:** Linear regression test for the IPL I-III, I-V and V/I ratio

		**Non-standard coefficient**	**Standard coefficient**	***P-value***	**95% confidence interval**	
					**Lower limit**	**Upper limit**
**IPL I-III right ear**	**Diabetic**	288/0	663/0	001/0>	203/0	373/0
	**Sex**	041/0	094/0	328/0	042/0-	124/0
	**age**	059/0	132/0	188/0	030/0-	149/0
**IPL I-III left ear**	**Diabetic**	217/0	637/0	001/0>	146/0	288/0
	**Sex**	008/0-	023/0-	823/0	077/0-	061/0
	**age**	033/0	094/0	372/0	041/0-	108/0
**V/I ratio right ear**	**Diabetic**	967/2	344/0	010/0	728/0	205/5
	**Sex**	748/0-	087/0-	495/0	930/2-	435/1
	**age**	928/1-	216/0-	105/0	276/4-	418/0
**V/I ratio left ear**	**Diabetic**	469/3	257/0	056/0	096/0-	034/7
	**Sex**	476/0-	035/0-	785/0	953/3-	00/3
	**age**	126/2	152/0	259/0	611/1-	863/5
**IPL I-V right ear**	**Diabetic**	079/0-	107/0-	446/0	287/0-	128/0
	**Sex**	046/0-	061/0-	653/0	248/0-	157/0
	**age**	019/0	024/0	863/0	198/0-	236/0
**IPL I-V left ear**	**Diabetic**	108/0-	137/0-	321/0	325/0-	108/0
	**Sex**	126/0-	159/0-	238/0	337/0-	086/0
	**age**	017/0-	021/0-	879/0	245/0-	210/0

IPL: interpeak latency

**Table 4:** Linear regression test for the IPL III-V and the PTA

		**Non-standard coefficient**	**Standard coefficient**	***P-value***	**95% confidence interval**	
					**Lower limit**	**Lower limit**
**IPL III-V right ear**	**Diabetic**	201/0-	292/0-	031/0	383/0-	019/0-
	**Sex**	107/0	155/0	230/0	070/0-	285/0
	**age**	002/0-	002/0-	987/0	192/0-	189/0
**IPL III-V left ear**	**Diabetic**	160/0-	237/0-	085/0	343/0-	023/0
	**Sex**	060/0	089/0	502/0	118/0-	239/0
	**age**	001/0>	001/0-	997/0	192/0-	191/0
**PTA right ear**	**Diabetic**	141/2	220/0	109/0	489/0-	771/4
	**Sex**	450/0	046/0	726/0	115/2-	015/3
	**age**	968/0	096/0	485/0	789/1-	726/3
**PTA left ear**	**Diabetic**	219/3	319/0	012/0	728/0	709/5
	**Sex**	941/0	093/0	441/0	488/1-	370/3
	**age**	679/2	256/0	045/0	068/0	290/5

IPL: interpeak latency, PTA: pure tone average.

**Table 5:** Relationship between the duration of diabetes and parameters of the ABR test and the PTA

**parameter**		**Less than 5 years** **(mean±SD)**	**More than 5 years** **(mean±SD)**	***P-value***
**Wave I**	**right**	181/0 ± 37/1	131/0 ± 42/1	409/0
	**left**	195/0 ± 38/1	205/0 ± 43/1	561/0
**Wave III**	**right**	231/0 ± 62/3	220/0 ± 67/3	539/0
	**left**	244/0 ± 61/3	224/0 ± 61/3	969/0
**Wave V**	**right**	356/0 ± 52/5	389/0 ± 48/5	798/0
	**left**	315/0 ± 57/5	374/0 ± 50/5	613/0
**IPL I-III**	**right**	273/0 ± 29/2	141/0 ± 22/2	325/0
	**left**	165/0 ± 20/2	178/0 ± 18/2	813/0
**V/I ratio**	**right**	83/6 ± 69/4	22/5 ± 21/4	830/0
	**left**	41/12 ± 90/6	52/5 ± 32/5	648/0
**IPL I-V**	**right**	693/0 ± 97/3	324/0 ± 05/4	689/0
	**left**	672/0 ± 01/4	433/0 ± 99/3	952/0
**IPL III-V**	**right**	606/0 ± 04/2	254/0 ± 84/1	225/0
	**left**	579/0 ± 09/2	285/0 ± 86/1	159/0
**PTA**	**right**	972/5 ± 72/12	061/7 ± 37/12	887/0
	**left**	073/6 ± 84/12	839/6 ± 89/12	984/0

IPL: interpeak latency, PTA: pure tone average, SD : Standard Deviation.

## Discussion

The ARB test is an essential tool for detecting any lesion of the auditory nerve and the brainstem among the diabetes patients (18). The results of the present study suggested that the absolute latency of I was significantly decreased in the diabetic patients’ group. On the other hand, the absolute latency of III and IPL I-III were higher in the diabetes patients than the control group. The IPL I-III is often considered a measure of the peripheral conduction time from the 8th nerve to near the cochlear nucleus and the results of the current study indicated disruption of this conduction time. These results coincided with the results of the study conducted by Kiran BS et al. (2022) that found the increased of the absolute latency of wave III and IPL of I-III waves in the diabetes patients group compared to the control group (19).

The absolute latency of III is produced by nerves near the cochlear nucleus and the increase in which indicates involvement at the surface of the brainstem. The cochlear nerve is innervated directly from the 8th nerve through the inner ear canal, which may account for the observed disturbance in the absolute latency of III. Neilson et al. (1966) and Makishima et al. (1971) showed the central neuropathy based on the degeneration of the brain and atrophy of the cochlear ganglion in diabetes patients. they concluded that microangiopathy of stria vascularis was a major cause of the central neuropathy in these patients (7, 20). Makishima et al. demonstrated that medial rotation of the cochlea was associated with demyelination of the eighth cranial nerve in diabetes patients (7).

The results of our study indicated no significant differences in the absolute latency of V and the IPL I-V between the two groups, but a significant relationship was observed between the I/V ratio and diabetes in the right ear. The wave V is generated by the nucleus of the inferior colliculus in the midbrain. Moreover, the current study found that the IPL III-V significantly increased in the right ear of diabetic patients. These findings revealed that the brain stem pathway from the superior olivary complex to the inferior colliculus was damaged in the right ear of the diabetic patients. 

In a study by Batham et al. (2017), the absolute latency of V and the IPL I-III were significantly higher in diabetic patients with duration of disease more than 5 years than in diabetic patients with duration of disease less than 5 years in the left ear. They found no significant relationship in the right ear (21). In our study no significant relationship was observed between the duration of diabetes more than 5 years and the parameters of the ABR test in both ears. Abo-Elfetoh et al. (2016) reported the elevation of the parameters in the ABR test among the diabetic patients compared with the control group. their findings confirmed the central neuropathy (22). Huang et al. (2010) and Al-Azzawi and Mirza (2004) found a significant increase in IPL I-III and I-V, but they did not observe a significant increase in IPL III-V. Their findings were similar to the results of our study. Involvement of the lower and upper brainstem in the diabetic patients is mainly associated with an increase in the central conduction time (23-25).

Bhattarai et al. (2016) reported that all parameters related to the ABR test except IPL III-V were higher in diabetic patients than in the control group (26). Akinpelu et al. (2014) observed that the absolute latency of V significantly increased in diabetic patients (27). Mahalik et al. (2014) reported an increase in all parameters of the ABR test. The increase in IPL III-V and absolute latency of I was bilateral and the increases in absolute latencies of III, V, IPL I-III and I-V were unilateral (8). Some previous studies have shown that the hearing impairment in diabetic patients is related to the duration of diabetes (28, 29). Baweja et al. (2013) found no significant relationship between the duration of the diabetes and the parameters of the ABR test (30). Samatra et al. (2020) reported significantly increased absolute latency of V in the diabetic patients with the duration of disease more than 5 years. The other parameters of the ABR test were also increased in the patients with longer duration of diabetes although this increase was not significant (31).

On the other hand, the patients in our study had a mean HbA1c value that was slightly higher and relatively good for diabetic patients compared with the control group, indicating that the patients had relatively good diabetes control. Sushil et al. (2016) reported that the absolute latency of III, IPL I- III, I-V, and III -V significantly increased in the diabetic patients with poorly controlled diabetes compared with the patients with well-controlled diabetes (32).

Samatra et al. (2020) demonstrated that patients with poorly controlled diabetes had more elevated the ABR parameters although this relationship was not significant (31). We found no significant difference in the absolute latency of V and the IPL I-V between the two groups in both ears, so it is possible that our diabetic patients did not have complete progression of the nerve damage because of the good glucose control and relatively moderate average duration of diabetes. The absolute latency of wave V is produced by the terminal nuclei of the central auditory nerve pathway, which may indicate that nerve damage progresses gradually from the primary to the terminal nucleus (30). In the current study we found the hearing threshold of diabetic patients was higher than the control group in both ears, but this finding was significantly higher only in the left ear. Mahalik et al. (2014) observed no significant difference in the PTA between the diabetic patients and control groups at all frequencies (8). Bhattarai et al. (2016) reported that the PTA in the diabetic patients’ group was significantly higher than the control group at all frequencies (26).

Akinpelu et al. (2014) demonstrated that the pure tone audiometry thresholds were higher in diabetic patients for all frequencies but were significantly more at 6000 and 8000 Hz. They observed that the PTA threshold was mostly below 30 dBHL in the type 2 diabetes, although it was slightly higher at higher frequencies. This means that the diabetic patients are less likely to be affected by hearing loss. However, these mild degrees of hearing loss may be exacerbated by other conditions that effect on the hearing. However, hearing thresholds at lower frequencies were found within the normal or mild range of hearing impairment in both the diabetic patients and control subjects and therefore may not have clinically significant effects (27). The results of the study showed that diabetes can involve the brain stem manifested in the ABR test including the absolute latency of wave III and IPL I-III and III-V. Our results indicated involvement of the central auditory system in diabetic patients. Signs of hearing threshold impairment were also noted in diabetic patients. The results of the present study point to the importance of performing hearing assessments in diabetic patients. 
